# Where did my dog go? A pilot study exploring the movement ecology of farm dogs

**DOI:** 10.3389/fvets.2023.1325609

**Published:** 2024-01-08

**Authors:** Kareemah Chopra, Gareth Enticott, Edward A. Codling

**Affiliations:** ^1^School of Mathematics, Statistics and Actuarial Science, University of Essex, Colchester, United Kingdom; ^2^School of Geography and Planning, University of Cardiff, Cardiff, United Kingdom

**Keywords:** farm dogs, global positioning system (GPS), animal movement, space-use, movement analysis

## Abstract

Movement ecology is important for advancing our comprehension of animal behavior, but its application is yet to be applied to farm dogs. This pilot study uses combined GPS and accelerometer technology to explore the spatial patterns and activity levels of free roaming farm dogs, *Canis familiaris* (*n* = 3). Space-use distributions and range sizes were determined to compare locations visited across days and between individuals, as well as in relation to specific areas of interest. Individual activity levels were analyzed and compared within and between dogs. Space-use patterns and range sizes showed variation among the dogs, although substantial similarity in overall spatial distributions were observed between each pair. Among the dogs, the extent of spatial distribution overlap between days varied, with some individuals exhibiting more overlap than others. The dogs allocated different amounts of their time close to landscape features, and to slow-, medium-, and fast movements. This study demonstrates the potential of using automated tracking technology to monitor space-use and interactions between dogs, livestock, and wildlife. By understanding and managing the free ranging behavior of their farm dogs, farmers could potentially take steps to improve the health and wellbeing of both their dogs and their livestock, limiting disease spread, and reducing the possibility of related economic losses.

## 1 Introduction

Movement ecology investigates the patterns and processes governing the movement of animals (1). By understanding movement patterns, we can gain valuable insights into animal ecology and behavior, including resource requirements (2, 3) and responses to environmental change (4, 5). Major technological advancements over the last decade are creating vast opportunities to collect data that cannot be obtained through direct observation alone (6). GPS (Global Positioning System) technology has revolutionized the study of animal movement, enabling the collection of detailed movement and location data, enhancing our understanding of habitat utilization and space-use (7). GPS has been successfully used in studies involving a diverse range of animals, from birds (8) to mammals (9). By harnessing this technology, we can analyze various aspects of animal movement on a fine-scale including migration routes (10, 11), foraging behavior (12, 13), and habitat selection (14, 15). In the context of farming, GPS collars have become widely and commercially available and have been used effectively to track livestock to inform herd management, including regrouping and pasture rotation (16–18).

One specific area of interest within movement ecology is the investigation of animal space-use, including the concept of a home range, as defined by Burt (19) as “the area transversed by the individual in its normal activities of food gathering, mating, and caring for young.” GPS-based studies typically estimate home range through calculating distributions and densities of space-use (20), enabling identification of core areas where animals spend a significant amount of time (21), and temporal shifts (22, 23). By analyzing home ranges, it is possible to better understand crucial aspects of an animal’s behavior, including territoriality (24), mating strategies (25), resource utilization (26), and responses to habitat fragmentation (21).

Working farm dogs (such as sheep dogs) conduct important work including herding and guarding livestock (27), as well as providing companionship to shepherds working in isolated conditions (28). Where farm dogs work frequently, they may be susceptible to health issues; a longitudinal study conducted in New Zealand revealed that 60% of working farm dogs developed at least one musculoskeletal abnormality (any physical sign regardless of severity) over 4 years (29). Health complications can greatly affect a working dog’s quality of life, through pain and limited mobility, and may also place an economic burden on farmers. To address these challenges, tracking technology such as GPS could help by continuously monitoring changes in speed or activity levels linked to physical and cognitive performance, e.g., dog activity patterns have been linked to fractional lifespan and working memory (30). Differences in space-use and activity in housed cattle have been linked to health conditions such as lameness (31), and similar approaches could potentially be used to monitor and detect health and welfare issues in working dogs. Interventions could then include workload adjustments or treatments, ultimately improving farm dog health and wellbeing.

Whilst working farm dogs are often supervised, at other times they may have freedom to roam around the farm or the surrounding countryside unsupervised, the extent to which is likely to be dependent on the farmer, their work, and the time of year (32). Implementing a user-friendly GPS interface could provide farmers with a valuable tool to ensure their (guardian) dogs are actively patrolling areas where livestock are kept, without venturing beyond farm boundaries (which could lead to theft) or entering hazardous areas. This becomes particularly important in areas where biohazards exist, since farm dogs may transmit pathogens to livestock (33). Research has shown links between various diseases in wild populations and dogs, such as heightened cases of rabies in jackals (*Canis aureus*) in the presence of domestic dogs in neighboring communities (34, 35), and increased exposure of numerous pathogens to African wild dogs through contact with domestic dogs (36). Using GPS to map out home ranges and understand the overlap in space-use between wild populations (dogs or other animals), livestock and working or domestic dogs could help minimize disease transfer risks.

Despite the known association between health, welfare and behavior, and the importance of working farm dogs within agriculture, there is little or no research on the use of automated tracking technology to monitor farm dogs. Continous monitoring could help farmers take steps to improve the health and wellbeing of their dogs and their livestock, and to reduce the likelihood of economic losses through the loss of labor and disease outbreaks. To address this gap in the literature, here we present the results of a pilot study that is the first to demonstrate how GPS tracking and movement ecology approaches can be used to explore the movement and space-use of three free-roaming farm dogs. In particular, we map and compare home ranges, determine space-use in relation to areas where pathogens may be present, and analyze and compare individual dog activity levels over time.

## 2 Methods

### 2.1 Study duration and site

Consent for the study was provided by a livestock farmer in mid Wales. The farm was a mixed farm with approximately 400 ewes and 100 cattle (cows and calves), occupying approximately 100 hectares.

**Figure 1:**
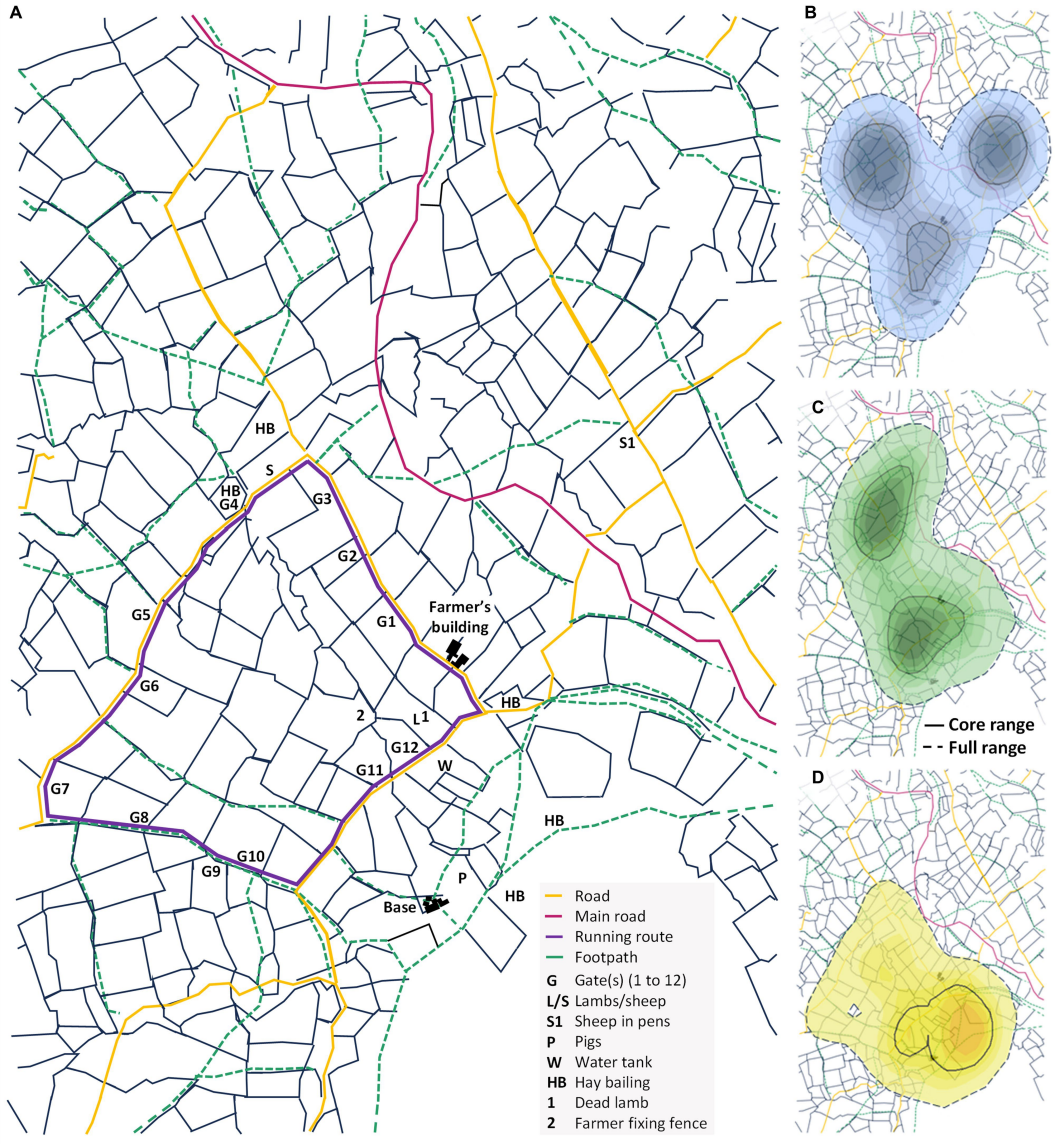
**(A)** The study site in mid-Wales. Areas of interest are marked as: gate(s) (G1-12), locations where sheep/lambs or pigs were present (S and P respectively), pens holding sheep (S1), and the water tank (W), areas used for hay bailing (HB), where the farmer found a dead lamb (1) and where the farmer was fixing a fence (2). The purple line indicates a running route used by the dogs’ owners. **(B–D)** Space-use distribution density, showing the positions of dogs **(B)** 1 (blue), **(C)** 2 (green) and **(D)** 3 (yellow) tracked using GPS. Kernel density is colored from low (light blue; 95%) to high (dark blue; 20%), with core range (50%) and full range (95%) contours in solid black lines and hashed black lines, respectively.

Data were collected from 17th September 2022 to 31st September 2022 and on 31st March 2023. Figure 1A shows the study site, including marked areas of interest such as gates (G1 to G12), footpaths and specific areas used during the study duration including the locations of livestock, a running route, and areas the farmer: bailed hay, fixed a fence and placed a water tank.

### 2.2 Animals and GPS sensors

Ethical approval was provided by the University of Essex ethics committee, reference numbers ETH2122-2165 and ETH2122-216. Five dogs were present on the farm including a male Huntaway-Collie cross and a female Labrador, and the remainder were used for the study based on discussions with the farmer regarding their daily lives and levels of activity. Dog 1 was a female pet Springer Spaniel (1.5 years old), dog 2 a working Huntaway-Collie cross (6 years old), and dog 3 a Huntaway in training (1.5 years old).

Commercial GPS sensors (Tractive GPS Tracker 4 for Dogs; accuracy up to 8 m with a clear line of sight) (37) were deployed on the free-roaming dogs via neck collars (*n* = 3 dogs during September 2022; dog 2 was tagged again in March 2023). The Tractive sensor is lightweight (35 g), waterproof, and is specifically designed for dogs, with dimensions of 71 × 28 × 17 mm and a battery life of up to 7 days. Positional data were set to record every 2 minutes while the dogs were moving (as determined by the sensor using the built-in accelerometer). The system includes a user-friendly interface that displays location in real time (and up to 365 days history), in contrast to previous studies using similar commercially available GPS collars, e.g., (38, 39). Hence this system was ideal for use in a participatory manner with farmers.

### 2.3 Pre-processing of GPS data

The total number of (x, y) positional data points collected were: 2449 for dog 1, 1743 for dog 2 (1,591 data points in September and 152 data points in March) and 928 for dog 3. We removed instances where the latitude and/or longitude coordinates were recorded as exactly the same for consecutive recordings, which was likely a result of the sensors switching off during low activity including when the farmer was charging them (in the evening/night after work shifts or when a battery died) (removing 33.34% of the original data). Instances where dogs were detected to be moving faster than 30 mph were also excluded (0.08% of original data; only four consecutive data points where the farmer confirmed the sensor was in the tractor). As we were interested in the movements of the dogs when they are free roaming away from the farm, rather than close to the farmer’s buildings, we excluded data points within a 100 m radius from the farmer’s base building and another building owned by the farmer where the dogs were kept (labeled ‘base’ and ‘farmer’s building’, respectively in Figure 1A) (1,500 and 983 data points were removed, respectively; 48.49% of the original data removed during this step). Lastly, we removed nonsensical data found by manually observing the daily trajectories of the dogs; only five consecutive data points were removed where the farmer had accidentally carried a sensor to the local town (0.10% of the original data removed during this step). A total of 921 data points (*n* = 3 dogs) remained for analysis. Refer to Supplementary material S1 for further details on these pre-processing steps.

### 2.4 Data visualization and home range

A range of approaches are available for estimating home range size (40). As our visualizations were created in part to provide a general overview of the data to the participant farmer, we estimate utility distribution (UD) using a bivariate kernel, a widely used and recommended method which smooths data to reduce noise (41, 42). For this, we use the “kernelUD()” function in the R package “adehabitatHR,” whereby the UD is estimated at the central point of each pixel within a grid (43). To ensure meaningful comparisons between utility distributions, we used a consistent grid configuration with virtual cells of 0.1 km x 0.1 km (longitude × latitude). We then computed kernel density contours at 20, 30, 50% (core range), 65, 80 and 95% (full range).

For completeness, we also plot the space-use distributions of the dogs’ including data points located close to the farm buildings (see Section 2.3 of Supplementary material).

### 2.5 Space-use comparisons

The Bhattacharyya coefficient (BC) measures the similarity between two probability distributions and was used to compare the space-use patterns of the dogs. The BC was calculated based on the UDs of the dogs’ space-use patterns (as determined in Section 2.4) (see equation below), for each pair of dogs (dogs 1 and 2, dogs 1 and 3 and dogs 2 and 3).


$$BC=\sum \left(\sqrt{P_{i}\times Q_{i}}\right)\text{,}$$

where *P_i_* and *Q_i_* are the normalized distributions of the two UDs being compared, and the sum is taken over all grid cells. A higher BC indicates a greater similarity in space-use, with 0 indicating no similarity and 1 indicating complete similarity.

We also compared the space-use patterns of each dog with its own history across all pairs of study days, with dog 1 being compared over 4 days, dog 2 over 9 days, and dog 3 over 7 days (i.e., for dog 1, we drew ten comparisons, between days 1 and 2, days 2 and 3 and days 1 and 3 etc.).

### 2.6 Analysis of landscape features

In this section of our analysis, we employ location data to quantify the proximity and overlap of individuals to specific points of interest. As an illustrative example, we examine the relative positioning of dogs to areas where direct or indirect interactions with other animals (wild or livestock) or people may occur: gates (labeled G1 to G12), footpaths and field boundaries (refer to Figure 1). Data points within a 25 m radius of a given landscape feature were defined as within close proximity and those within 250 m radius were considered to be in the general surrounding area. For each dog, we counted the number of data points located in each of these defined areas over the entire study duration, and we calculated the total time each individual was located within them across the study duration. Fixes recorded more than 1 hour apart were excluded from the time calculations (one data point for dog 2 and 15 data points for dog 3).

We defined footpaths from those marked as such on Ordnance Survey maps, including bridleways and National Trails/long distance routes (see in Figure 1). We manually found the geographic coordinates of each footpath, including the starting point, each point where the route changed direction, and the end point. This approach, while effectively providing detailed coordinates, also inadvertently smoothed the footpaths (although differences in the number of data points located within our defined radii around the footpaths would be negligible). Using the R package “sp” (44), we converted the coordinates for each footpath into a spatial object using the “Line()” and SpatialLines() functions. We also converted all coordinate data for each given dog into a spatial object using these functions. For each dog, the gDistance() function from the “rgeos” R package (45) was used to calculate the shortest distance between each location point and each footpath. A similar method was used to measure proximity to gates and field boundaries (focusing on only 25 m radius for the latter due to closeness of the fields).

### 2.7 Activity levels

To provide further insight into the behavioral patterns of the dogs, we calculated movement between each data point for each dog (distance traveled divided by time elapsed between successive GPS fixes) Again, fixes recorded more than 1 hour apart were not included in the calculations (16 data points in total). We compared average movements across the entire study duration between the dogs. Movements were then classified into three categories: slow (< 50 meters/min [m/m]), medium (≥ 50 m/m and < 100 m/m) and fast movements (≥ 100 m/m) and we compared the number of data points in each of these categories between the dogs. Average movements were also compared for dog 2 between September 2022 and March 2023.

## 3 Results

### 3.1 Space-use distribution

All dogs were frequently located in areas where hay bailing took place, as well as in fields near the farmer’s base building (Figure 1). Dog 1 was also frequently located in an area where sheep were held in pens (Figures 1A,B). It appears that dog 3 had the smallest core and full range size (0.54 km^2^ and 2.72 km^2^ respectively) compared to dog 1 (0.89 km^2^ and 3.38 km^2^ respectively) and dog 2 (0.77 km^2^ and 3.06 km^2^ respectively). There is a substantial degree of overlap in space utilization between all the dogs, with a Bhattacharyya coefficient [BC] of 0.82 between dog 1 and 2, 0.65 between dog 1 and 3, and 0.77 between dog 2 and 3 (Supplementary Table S1).

**Figure 2:**
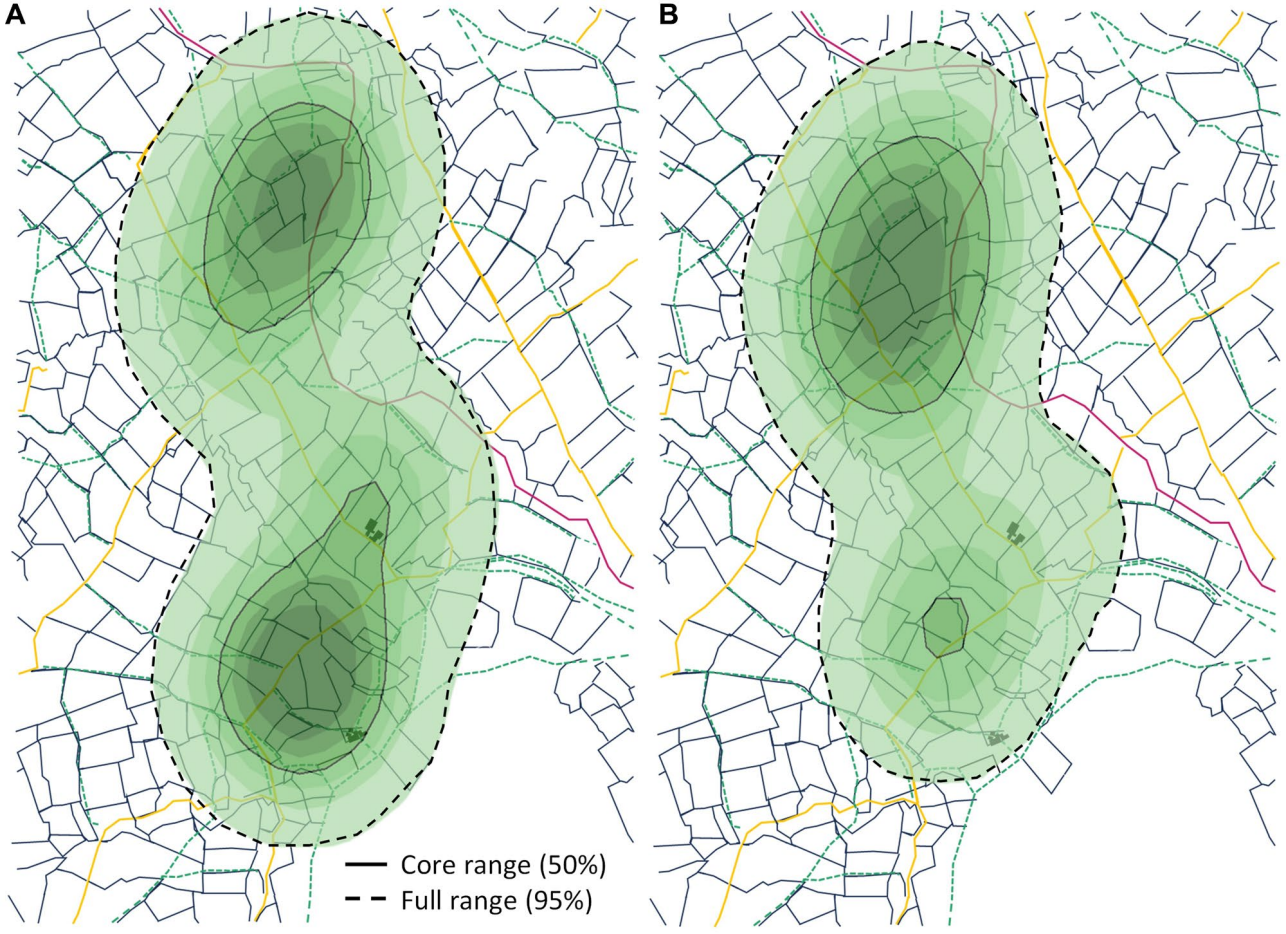
Space-use distribution density, showing the positions of dog 2 on 2 days with high similarity (Bhattacharyya coefficient = 0.94): **(A)** 28/09/2022 and **(B)** 31/03/2023. Density is measured on a scale from low (light blue) to high (dark blue), with core range (50%) and full range (95%) contours marked as solid gray lines and hashed black lines, respectively.

Dog 1 shows the least overlap in space utilization between days, with a mean BC of 0.27 (range of BC = 0.09 to 0.63) compared to dog 2 (mean BC of 0.67; range of BC = 0.13 to 0.94) and dog 3 (mean BC = 0.54; range of BC = 0.21 to 0.84) (Supplementary Tables S2–S4). Figure 2 shows examples of daily spatial distributions for dog 2, where the BC is calculated to be the highest at 0.94, which is interestingly on a day in September and a day in March; we are not aware of any specific reasons for the dog behaving similarly on these days.

### 3.2 Proximity to landscape features

**Figure 3:**
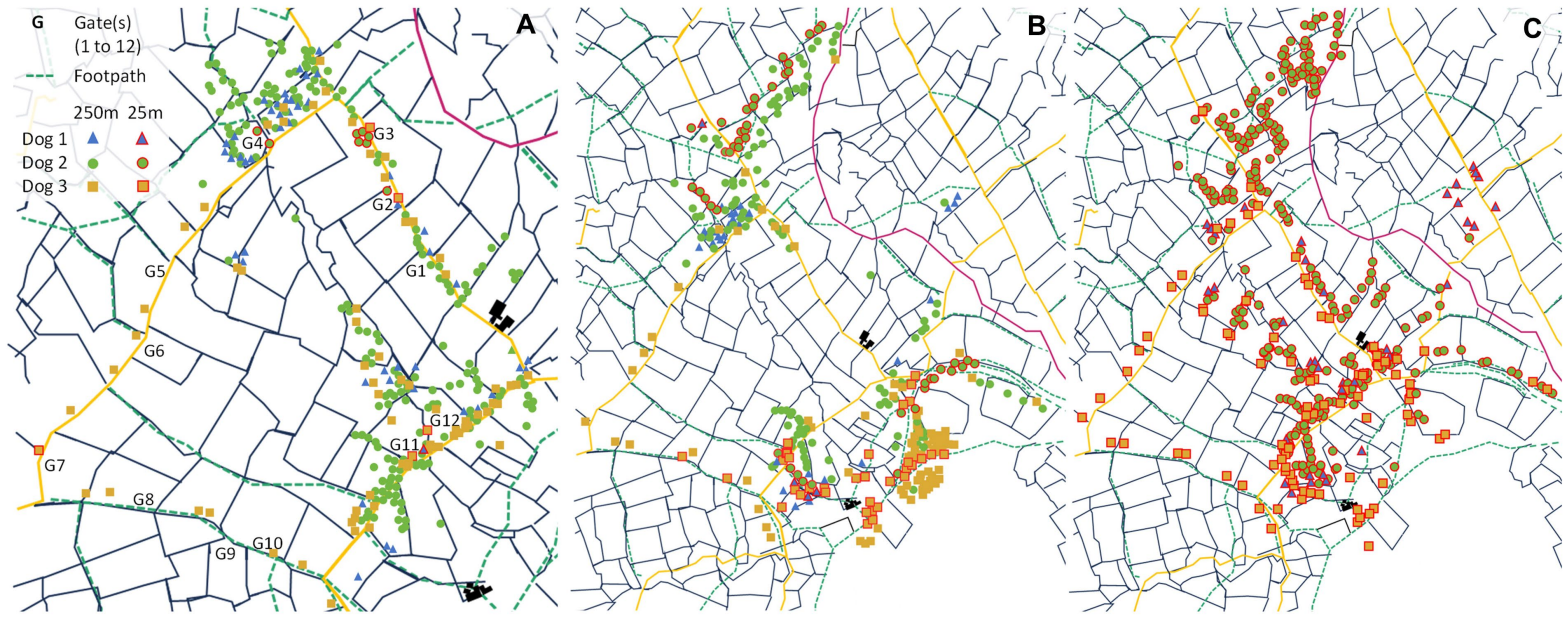
The spatial distribution of dogs over the entire study duration in relation to **(A)** gates (G1 to G12), **(B)** footpaths (green hashed lines), and **(C)** field boundaries (black lines). Dog 1 is represented by blue triangles, dog 2 by green circles, and dog 3 by yellow squares. All data points located within 250 m of these landscape features represented without borders, with those within 25 m radius are bordered in red. Note that only data points within 25 m of the field boundaries **(C)** are shown for the purposes of the analysis, and the map for **(A)** is zoomed in on the area with the gates to help differentiate between the data points.

The gates that the dogs were located around were primarily near the farmer’s buildings (gates 11 and 12; Figure 3A; refer to Figure 1A). The footpaths the dogs were detected within the general area of were primarily around the base building and gate 4 (Figure 3B; refer to Figure 1A). The dogs were only detected within close proximity (25 m radius) of any of the gates on a few occasions (one, eight and five data points for dogs 1, 2 and 3 respectively) and they were occasionally detected close to the footpaths (four, 57 and 52 data points for dogs 1, 2 and 3 respectively) (Figure 3B). Interestingly, the dogs were frequently located within close proximity to the field boundaries (71, 70 and 60% of time across the study duration for dogs 1, 2 and 3 respectively; Figure 3C). Refer to Section 2.3 of the Supplementary material for further details.

### 3.3 Activity levels

**Figure 4:**
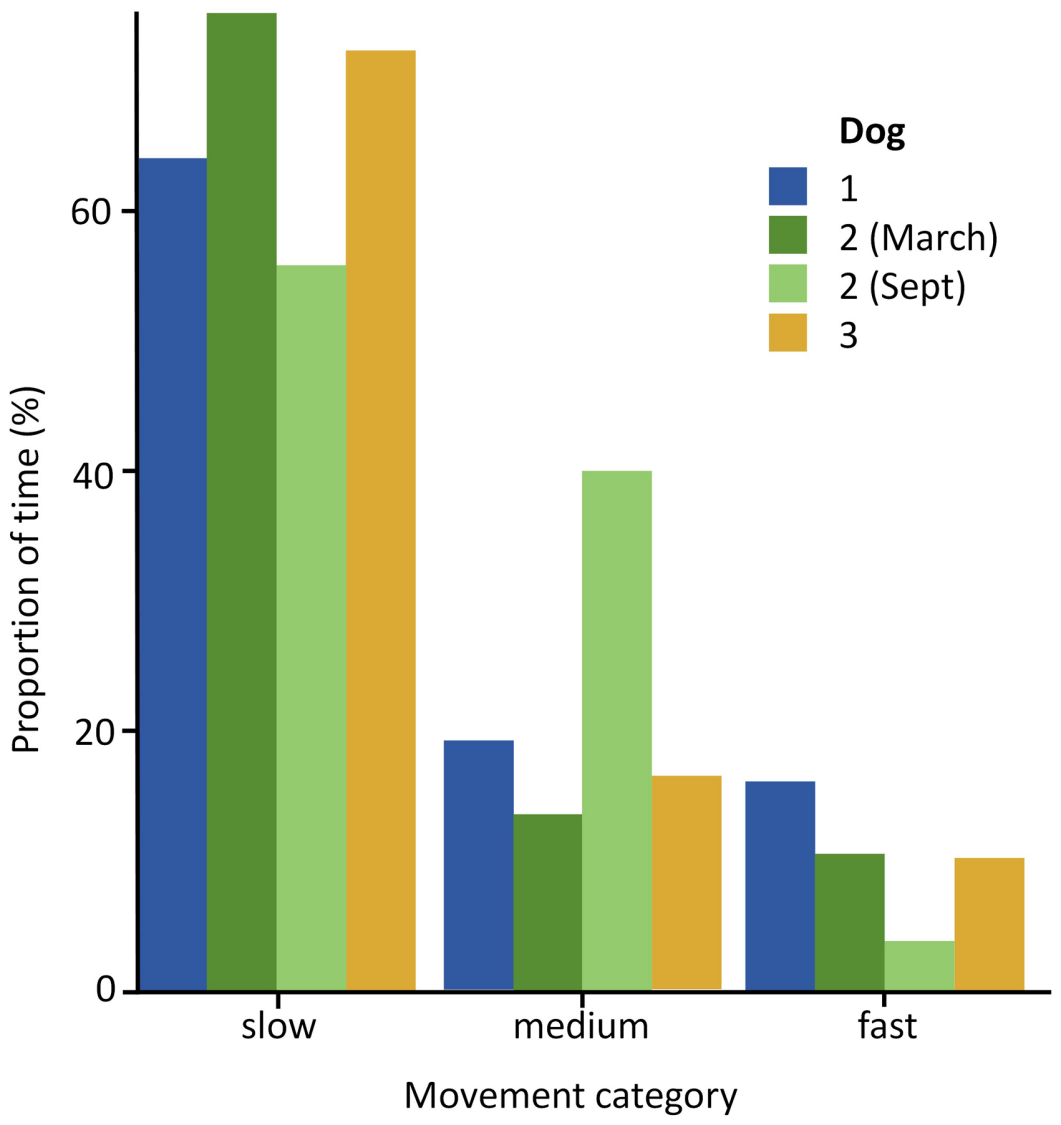
Comparison of proportion of time dedicated to: slow (< 50 meters/min [m/m]), medium (≥ 193 50 m/m and < 100 m/m) and fast movements (≥ 100 m/m) across the full study period (September 2022 and, for dog 2 only- March 2023). Time budgets are shown for each of the dogs: labeled dog one (*n* = 111 data points), dog two (*n* = 547 data points) and dog three (*n* = 263 data points), colored blue, green [light for September 2022 and dark for March 2023] and yellow, respectively. Note that the number of data points is not equivalent to time, as the duration between each fix was not consistent.

The dogs exhibited varying behavioral patterns, with dog 1 spending notably less time on slow movements (64%) compared to dogs 2 and 3 (74 and 72% respectively) over the full study period (Figure 4). Additionally, dog 1 spent more time on fast movements (16%) compared to dogs 2 and 3 (11 and 11% respectively) over the full study period (Figure 4). On average, across the entire study duration, movements of 50.90 meters/min (m/m) were calculated for dog 1, 48.66 m/m for dog 2, and 51.09 m/m for dog 3 (Figure 4). Dog 2 exhibited a faster average movement in March (54.36 m/m) than in September (48.42 m/m), while also spending less time on slow movements during March (56%) compared to September (75%) (Figure 4).

## 4 Discussion

This pilot study has highlighted how automated animal-mounted tracking technology such as GPS collars can provide valuable insights into the movement patterns, space-use, and activity of free-roaming dogs on farms. Given the very small sample size (*n* = three dogs) our study should be considered more of an illustrative proof of concept. Nevertheless, our results indicate that farm dogs can exhibit distinct space-use patterns which vary temporally, with differences in home range size and distribution, proximity to areas of interest, and activity levels. Behavioral differences, measured within and between dogs over time, could allow farmers to monitor the health, welfare, and working performance of their dogs in an automated way. Meanwhile, better understanding of dog ranging behavior, space-use patterns, and proximity to landscape features that may act as potential pathogen hotspots, could potentially enable farmers to improve farm biosecurity and livestock management.

In this study the three farm dogs tended to position themselves in specific areas which varied between the individuals but were generally associated with routine farming activities. For example, all three dogs were frequently observed where the farmer bailed hay, and dog 1 was frequently located where sheep were herded and kept in pens (Figure 1). Of particular interest, dog 1 exhibited the most extensive core and full range (0.89 km^2^ and 3.42 km^2^ respectively) compared to dog 2 (0.77 km^2^ and 3.06 km^2^ respectively) and dog 3 (0.54 km^2^ and 2.72 km^2^ respectively) (Figures 1B–D). Dog 1 was not a working dog, so may have had more freedom to roam, compared to the other study dogs. It is well-established that domestic dogs exhibit individual differences in space-use and activity patterns (38, 46, 47). Space utilization is influenced by factors such as body condition, age and breed (48–51). For instance, older dogs may experience limited mobility (48, 52), or may instead have extended, and more established, home ranges due to dominance (46). Also, poor body score condition has been associated with smaller home range size (48), although, this finding also appears inconsistent, potentially due to food availability impacting home range (53). In addition to resources, space utilization and the crossing of boundaries may be impacted by the presence of other animals, such as livestock or other dogs. Space utilization may also be influenced by an individual’s life history; for instance, research indicates that older livestock guarding dogs tend to associate more with their flock compared to their younger counterparts (54). Our findings serve as a valuable starting point for future studies to investigate the extent to which farm dogs space-use and movement behavior is a matter of choice or whether it is more influenced by farmer activities.

The three farm dogs appeared to spend various amounts of time near landscape features, possible high-traffic areas, with a noticeable tendency to position near gates and footpaths closest to the farmer’s buildings (Figure 3). The dogs were only detected close to (25 m radius) any gates or footpaths on a few occasions (Supplementary Tables S5, S6). However, the dogs were frequently detected close to the field boundaries, particularly dogs 1 and 2, with 71, 70 and 60% of total time across the study duration spent within a 25 m radius for dogs 1, 2 and 3, respectively, (Figure 3; Supplementary Table S6). A previous study found that domestic dogs have a preference for anthropogenic areas including roads (55), but this may vary on an individual basis. It may therefore be necessary to consider restricting the movement of specific dogs around certain high-traffic landscape features to mitigate the risk of disease transmission between wildlife and livestock.

Preferences for specific areas among free-roaming dogs are likely to exhibit temporal variability, as evidenced by the diverse levels of similarity in daily space-use observed in our study. We observed the most similarity in space-use between days for dog 2 (mean BC of 0.67 across all days; Figure 1C) and the least similarity between days for dog 1 (mean BC of 0.27 across all day; Figure 1B). This may be because dog 2 was an experienced working dog, and work may have been situated in similar locations, whereas dog 1 was a pet dog with a greater ability to roam further. It is possible that environmental changes such as weather conditions could have influenced the dogs’ spatial behavior. For instance, studies have indicated that dogs roam further in the dry season compared to the wet season in the Torres Strait and a Northern Peninsula Area of Australia (47, 56). More extensive roaming may increase the risk of disease transmission between dogs, livestock, and wildlife. Furthermore, during lambing season, working dogs may be more inclined to stay close to livestock, which may have implications for disease spread.

The three dogs spent most of the study exhibiting low activity, with a gradual decline in fast movements (Figure 4). This is expected, considering the typical behavior of domestic dogs (57–59). Dog 1 stood out as spending the most time on fast movements in the September study period (Figure 4). Given dog 1 was a young pet Springer Spaniel, this may be accounted to higher energy levels. Conversely, the remaining dogs were Huntaways, either working (dog 3) or in training (dog 2), and they may have balanced their workload by engaging in less active behaviors, which supports our findings of their less extensive range sizes and higher similarity in their daily spatial distributions compared to dog 1. Alternatively, their less active behaviors may indicate more laid-back temperaments. Interestingly, in March, dog 2 spent less time on low active behaviors (56%) than during September (75%) (Figure 4). This might have been influenced by changes in workload or environmental conditions. Observations such as these could be employed to assess dog health by monitoring activity over time in the context of external factors. For example, lower activity or a slower than usual speed might be an indicator of lameness, while an increase in low active behaviors might signal a negative emotional state (60). Activity assessments could also contribute to ensuring safety on farms, such as helping prevent startling livestock. Additionally, a dog’s activity could be monitored during training to tailor training methods to suit the specific needs and progress of individuals effectively.

This study is intended as a pilot and included only a small sample size of three dogs; further investigation on a range of other farms with more, and different, dogs is required before any general conclusions can be drawn. Methodological innovations necessary for future studies include addressing the technical limitations of sensors, notably the GPS accuracy (up to 8 m) and battery life, the latter of which resulted in some missing data due to charging logistics. Future studies could employ custom GPS sensors with improved battery life, albeit at higher costs (61). To build on this pilot study and enhance our understanding of the free-roaming behavior of farm dogs, further research could involve a comparative analysis of the spatial patterns between dogs and farmers, possibly by equipping farmers with GPS sensors. Conducting longitudinal studies to investigate how space-use patterns may change over time, and according to individual and environmental factors, could further provide valuable insights for effective management. Another promising avenue of research lies in examining interactions between dogs, livestock, and wildlife. Such research would be particularly useful in resource-poor regions where free-roaming dogs, such as those carrying rabies (62), pose significant health risks.

## Data availability statement

The datasets presented in this article are not readily available because the raw data that support the findings of this study are available on request from the corresponding author, KC. The data are not publicly available due to the inclusion of information that could compromise the privacy of research participants. Requests to access the datasets should be directed to KC, km19088@essex.ac.uk.

## Ethics statement

The animal studies were approved by the University of Essex ethics committee, reference numbers 100 ETH2122-2165 and ETH2122-216. The studies were conducted in accordance with the local legislation and institutional requirements. Written informed consent was obtained from the owners for the participation of their animals in this study.

## Author contributions

KC: Writing – original draft, Writing – review & editing, Data curation, Formal analysis, Methodology. GE: Data curation, Funding acquisition, Methodology, Writing – original draft, Writing – review & editing. EC: Data curation, Formal analysis, Funding acquisition, Methodology, Writing – original draft, Writing – review & editing.
