# Supplementary material for: Where did my dog go? A pilot study exploring the movement ecology of farm dogs
Source: Front Vet Sci. 2024 Jan 8;10:1325609. doi: 10.3389/fvets.2023.1325609 (PMC10800614; doi:10.3389/fvets.2023.1325609)
Supplement: Supplementary file 1 [file Data_Sheet_1.docx]

Supplementary Material

# Pre-processing of location data

There was extended interruption because of a system malfunction on four of the study days for dog 1 (18/09/2022, 20/09/2022 and 21/09/2022, 28/09/2022) and seven of the days for dog 3 (19/09/2022, 20/09/2022, 23/09/2022, 25/09/2022, 26/09/2022, 28/09/2022 and 29/09/2022); data were not recorded on these days so could not be included in the analysis. In total 5120 data points were collected: 2449 data points for dog 1, 1743 data points for dog 2 (1591 in September and 152 in March) and 928 data points for dog 3.

To ensure the data were sensical before conducting the analysis, we pre-processed the data. Firstly, we removed instances where the latitude and/or longitude coordinates were recorded as exactly same for consecutive recordings (Fig A1). It is important to note that this included correct location data during periods during which the sensor was charging, hence stationary. This could occur when the recordings were a few minutes or up to an hour apart. This resulted in the removal of 33.34 % of the original data (1707 data points; step 1 in Supplementary Figure 1).

In the second stage of pre-processing, we removed instances where the sensors detected movements exceeding 30 mph. This filter was based on the maximum speed of the greyhound, the fastest breed of dog (American Kennel Club, n.d.; Wilcox, 2011). By setting this speed threshold, we aimed to eliminate any data points that may have been caused by factors other than the natural movements of the dogs, such as transportation on a vehicle. To calculate the speed of the farm dogs, we used a simple formula that involved measuring the distance between two points and dividing this by the time interval between recordings. There were only a few instances of this (four data points for dog 2), resulting in the removal of 0.08 % of the original data, and the farmer confirmed the dog was with them in the tractor.

We then excluded correct locations that were recorded within a fixed radius of 100 m from the farm base building to better understand the differences in space-use when dogs were free ranging beyond the immediate area surrounding the base building (refer to Fig 1 in main paper). Similarly, locations recorded within 100 m of another farm building were also excluded (refer to Fig 1 in main paper). It is important to note that these locations were likely to be correct and largely encompassed periods during which the dogs were resting. As a result, 1500 data points were lost for the base building and 983 data points were lost for the other farm building (total of 48.49 % of the original data removed; step three in Supplementary Figure 1).

Lastly, we manually observed the daily trajectories of each dog to check for unrealistic movements. There were five consecutive recordings whereby dog 3 was located to be in town, where the farmer had carried the sensor to the local town, and these were removed for analysis (0.10% of original data; step 4 in Supplementary Figure 2). As a result of the four data pre-processing steps, a total of 921 data points remained for analysis (111 data points for dog 1, 547 for dog 2 and 263 for dog 3).

**Supplementary Figure 1.** Pre-processing steps carried out on the data collected across the entire study duration (*n* = 3 dogs on 16^th^ September 2022 to 29^th^ September 2022 and *n* = one dog on 31^st^ March 2023) before data analysis: step 1, an automated check for data points recorded as exactly the same for consecutive recordings, step 2, removal of unrealistic speeds > 30 miles per hour, step 3, the removal of data points recorded within 100m of the main two farm buildings and step 4, a manual check of daily trajectories.

# Space-use distribution

Here we report detailed results from the space-use comparisons to further Section 3.1 of the main paper, using the Bhattacharyya coefficient as a measure of similarity (see Section 2.5 of Methods in main paper).

**2.1. Overlap between dogs**

There is a substantial degree of overlap in space utilization between all the dogs, ranging from Bhattacharyya coefficient [BC] of 0.65 to 0.82 (Table S2.1).

**Table 2.1.** Overlap of overall space-use (density distribution) across the study duration between dogs (1, 2 and 3) according to the Bhattacharyya coefficient. Lower correlation values are shown in lighter shades whereas darker shades indicate higher correlation values.

| **Dog** | **1** | **2** | **3** |
| --- | --- | --- | --- |
| **1** | - | 0.82 | 0.65 |
| **2** | 0.82 | - | 0.77 |
| **3** | 0.65 | 0.77 | - |

**2.2. Overlap between days**

Dog 1 shows varying overlap in space utilization between days, with a mean BC of 0.27 (range of BC = 0.09 to 0.63; Table S2.2), as does dog 3 with a mean BC = 0.54 (range of BC = 0.21 to 0.84; Table S2.4). Dog 2 shows the greatest mean degree of overlap between days with a mean BC of 0.67 (range of BC = 0.13 to 0.94; Table S2.3).

**Table 2.2.** Overlap of space-use (density distribution) according to the Bhattacharyya coefficient between study days for dog 1. Note that a comparison could not be drawn between 23/09/2022 and the remaining days, as only two data points were collected on this day.

| **Day** | **17/09/22** | **22/09/22** | **24/09/22** | **29/09/22** |
| --- | --- | --- | --- | --- |
| **17/09/22** | - | 0.16 | 0.63 | 0.09 |
| **22/09/22** | 0.16 | - | 0.38 | 0.13 |
| **24/09/22** | 0.63 | 0.38 | - | 0.22 |
| **29/09/22** | 0.09 | 0.13 | 0.22 | - |

**Table 2.3.** Overlap of space-use (density distribution) according to the Bhattacharyya coefficient between study days for dog 2.

| **Day** | **17/09/22** | **18/09/22** | **20/09/22** | **21/09/22** | **22/09/22** | **24/09/22** | **25/03/22** | **28/09/22** | **31/03/2023** |
| --- | --- | --- | --- | --- | --- | --- | --- | --- | --- |
| **17/09/22** | - | 0.86 | 0.33 | 0.54 | 0.75 | 0.87 | 0.75 | 0.68 | 0.75 |
| **18/09/22** | 0.86 | - | 0.39 | 0.55 | 0.79 | 0.91 | 0.57 | 0.79 | 0.80 |
| **20/09/22** | 0.33 | 0.39 | - | 0.13 | 0.49 | 0.39 | 0.49 | 0.46 | 0.34 |
| **21/09/22** | 0.54 | 0.55 | 0.13 | - | 0.63 | 0.67 | 0.48 | 0.65 | 0.71 |
| **22/09/22** | 0.75 | 0.79 | 0.49 | 0.63 | - | 0.93 | 0.79 | 0.92 | 0.93 |
| **24/09/22** | 0.87 | 0.91 | 0.39 | 0.67 | 0.93 | - | 0.80 | 0.87 | 0.91 |
| **25/09/22** | 0.75 | 0.57 | 0.49 | 0.48 | 0.79 | 0.80 | - | 0.62 | 0.68 |
| **28/09/22** | 0.68 | 0.79 | 0.46 | 0.65 | 0.92 | 0.87 | 0.62 | - | 0.94 |
| **31/03/23** | 0.75 | 0.80 | 0.34 | 0.71 | 0.93 | 0.91 | 0.68 | 0.94 | - |

**Table 2.4.** Overlap of space-use (density distribution) according to the Bhattacharyya coefficient between study days for dog 3.

| **Day** | **17/09/22** | **18/09/22** | **21/09/22** | **22/09/22** | **24/09/22** | **27/09/22** |
| --- | --- | --- | --- | --- | --- | --- |
| **17/09/22** | - | 0.59 | 0.80 | 0.48 | 0.79 | 0.64 |
| **18/09/22** | 0.59 | - | 0.27 | 0.29 | 0.21 | 0.26 |
| **21/09/22** | 0.80 | 0.27 | - | 0.48 | 0.84 | 0.60 |
| **22/09/22** | 0.48 | 0.29 | 0.48 | - | 0.39 | 0.75 |
| **24/09/22** | 0.79 | 0.21 | 0.84 | 0.39 | - | 0.70 |
| **27/09/22** | 0.64 | 0.26 | 0.60 | 0.75 | 0.70 | - |

**2.3. Closeness to features**

The dogs were frequently detected within the general area of the gates (250 m; dog 1 = 97 data points, dog 2 = 431 data points and dog 3 = 136 data points; Table S2.5). Out of all the gates, the dogs were located most often around: gate 4 (dog 1 = 36 data points and dog 2 = 75 data points and dog 3 = 6 data points within 250 m radius), gate 11 (dog 2 = 123 data points and dog 3 = 37 data points within 250 m radius) and gate 12 (dog 2 = 123 data points and dog 3 = 35 data points within 250 m radius) (Table S2.5). The dogs were also often detected within the general area of the footpaths (49, 317 and 193 data points for dog 1, 2 and 3 respectively; Table S2.6). Their presence within close proximity (25m radius) to the gates (total of one, eight and five data points for dogs 1, 2 and 3 respectively; Table S2.5) and footpaths (total of four, 57 and 52 data points for dogs 1, 2 and 3 respectively; Table S2.5-6) was limited. However, the dogs were frequently located close to the field boundaries (78, 369 and 118 data points for dogs 1, 2 and 3 respectively), considering the total number of data points collected per dog (111, 547 and 263 data points for dogs 1, 2 and 3 respectively; Table S2.6).

**Table 2.5.** The number of data points (and percentage of time across study period) located within close proximity to (a) given gate(s) (25 m) in comparison to the time spent within the general surrounding area (250m), for dogs 1 (total *n* = 111 data points), 2 (total *n* = 547 data points) and 3 (total *n* = 263 data points). The number of gates are provided. Note that the number of data points is not equivalent to the time spent near the gates, as the duration between each fix was not consistent.

| **Dog** | **Gate(s) (G) ID** | **Number of gates** | **Data points within**  **x radius of the gate [% of total]** | |
| --- | --- | --- | --- | --- |
|  |  |  | 25m | 250m |
| 1 | 1 | 3 | 0 [0%] | 4 [3.58 %] |
|  | 2 | 1 |  | 3 [2.69 %] |
|  | 3 | 4 |  | 26 [23.30 %] |
|  | 4 | 3 |  | 36 [32.25 %] |
|  | 5 | 1 |  | 2 [1.77 %] |
|  | 6 | 3 |  | 0 [0%] |
|  | 7 | 1 |  |  |
|  | 8 | 2 |  |  |
|  | 9 | 1 |  |  |
|  | 10 | 3 |  |  |
|  | 11 | 3 | 1 [1.09 %] | 13 [11.76 %] |
|  | 12 | 3 | 0 [0%] | 13 [11.82 %] |
| 2 | 1 | 3 |  | 31 [5.08 %] |
|  | 2 | 1 | 1 [0.17 %] | 20 [3.25 %] |
|  | 3 | 4 | 5 [0.69 %] | 58 [9,59 %] |
|  | 4 | 3 | 2 [0.34 %] | 75 [13.31 %] |
|  | 5 | 1 | 0 [0%] | 1 [0.17 %] |
|  | 6 | 3 |  | 0 [0%] |
|  | 7 | 1 |  |  |
|  | 8 | 2 |  |  |
|  | 9 | 1 |  |  |
|  | 10 | 3 |  |  |
|  | 11 | 3 |  | 123 [20.79 %] |
|  | 12 | 3 |  | 123 [20.73 %] |
| 3 | 1 | 3 |  | 9 [2.93 %] |
|  | 2 | 1 | 1 [ 0.37 %] | 8 [2.72 %] |
|  | 3 | 4 | 1 [ 0.34 %] | 11 [3.99 %] |
|  | 4 | 3 | 0 [0%] | 6 [2.19 %] |
|  | 5 | 1 |  | 7 [2.55 %] |
|  | 6 | 3 |  | 4 [1.45 %] |
|  | 7 | 1 | 1 [0.37 %] | 4 [1.47 %] |
|  | 8 | 2 | 0 [0%] | 4 [1.25 %] |
|  | 9 | 1 |  | 4 [1.27 %] |
|  | 10 | 3 |  | 7 [2.04 %] |
|  | 11 | 3 | 1 [0.37 %] | 37 [14.09 %] |
|  | 12 | 3 | 1 [0.38 %] | 35 [15.91 %] |

**Table 2.6.** The number of data points (and percentage of time across study period) located in the areas around footpaths (25 m and 250 m) and field boundaries (25 m) for dogs 1 (*n* = 111 total data points), 2 (*n* = 547 total data points) and 3 (*n* = 263 total data points). Note that the number of data points is not equivalent to the time spent near the landscape features, as the duration between each fix was not consistent.

| **Dog** | **Data points located within**  **x m radius [% of total time]** | | |
| --- | --- | --- | --- |
|  | **Footpaths** | | **Field boundaries** |
|  | 25m | 250m | 25m |
| 1 | 4 [3.58 %] | 49 [43.24 %] | 78 [70.64 %] |
| 2 | 57 [11.23 %] | 317 [55.82 %] | 369 [69.54 %] |
| 3 | 52 [22.70 %] | 193 [73.99 %] | 118 [60.46 %] |

**2.3. Including barn buildings**

In the main paper, we focus on the dogs’ space-use beyond the farm buildings, given these periods are when it is more difficult for farmers to observe their dogs’ movements. For completeness, in this section we show the space-use distributions for each dog without excluding locations within a 100 m radius of the two farm buildings. Data points corresponding to when the sensors were charging in the farm buildings (primarily during the late evening and night) were previously removed in step 1 of pre-processing, where ‘stuck’ sensors were removed, so no further data removal was required.

As expected, Supplementary Figure 2 highlights that the dogs spent most of their time in areas surrounding the farm buildings, which skews the space-use distributions heavily toward these areas (approximately half of the total original data points were located in these areas as noted in the Section 1).

**Supplementary Figure 2.** Space-use distribution density, showing the positions of dogs (A) 1 (blue), (B) 2 (green) and (C) 3 (yellow) tracked using GPS. Data surrounding the farmers buildings are included. Kernel density is colored from low (light blue; 95%) to high (dark blue; 20%), with core range (50%) and full range (95%) contours in solid black lines and hashed black lines, respectively.
